# Environmental sustainability assessment of tropical dairy buffalo farming vis-a-vis sustainable feed replacement strategy

**DOI:** 10.1038/s41598-019-53378-w

**Published:** 2019-11-14

**Authors:** P. Ravi Kanth Reddy, D. Srinivasa Kumar, E. Raghava Rao, Ch. Venkata Seshiah, K. Sateesh, K. Ananda Rao, Y. Pradeep Kumar Reddy, Iqbal Hyder

**Affiliations:** 1 Livestock Farm Complex, CVSc, SVVU, Proddutur, 516 360 India; 2 Department of Animal Nutrition, NTRCVSc, SVVU, Gannavaram, 521 102 India; 3 Administrative building, Sri Venkateswara Veterinary University, Tirupati, 517 502 India; 4 Livestock Farm Complex, NTRCVSc, SVVU, Gannavaram, 521 102 India; 5 AH Polytechnic college, SVVU, Banavasi, 518 323 India; 6 Buffalo Research Station, SVVU, Venkataramannagudem, 534 101 India; 7 Centre for Continuing Veterinary Education and Communication, CVSc, Sri Venkateswara Veterinary University, Tirupati, 517 502 India; 8 Department of Veterinary Physiology, NTRCVSc, SVVU, Gannavaram, 521 102 India

**Keywords:** Animal physiology, Environmental impact

## Abstract

Feeding management in dairy animals is crucial for ensuring optimal production apart from making the farming as a whole, a more sustainable activity. In our study we evaluated the production and environmental effects of two different feeding regimens i.e., one dominated by traditional cottonseed meal (CSM) and other with coated urea (slow release urea - SRU) as a replacement for CSM on dairy buffalo production. The SRU at 2% level was evaluated by conducting two different trials using twelve lactating Murrah buffaloes and four adult Murrah buffalo bulls. Neither diet nor dry period management showed any substantial effect on milk components, intakes, nutrients’ digestibility coefficients, and nutritive values. The SRU diet revealed increased (P < 0.01) rumen pH, ammonia nitrogen, volatile fatty acids, and microbial nitrogen yield, which were interacted with time of post-prandial sampling. The dynamics of nitrogen metabolism revealed unaltered N-based parameters, except for degradable-N intake and serum urea-N at 3 hr post-feeding. The CSM replacements did not influence (P > 0.05) the residual feed intake, but led to an enhanced milk retention efficiency of nitrogen, calcium, and phosphorus contents, thus reducing their impact on soil pollution and eutrophication of water bodies. Despite an unaltered (P > 0.05) enteric methane emission, SRU diets achieved in decreasing manure methane and nitrous oxide emission. Furthermore, the virtual water flow and lifecycle assessment revealed a water sparing effect and low carbon foot print per unit milk production. In summary, the CSM replacements with SRU could achieve an economical and eco-friendly production system from animal nutrition perspective.

## Introduction

Amidst the growing advocacy for sustainable development, there is also a need to improve the environmental footprints of dairy farming. It is being predicted that the demand for animal products is expected to increase further by 70% by 2050^1^, which may exert lot of pressure on the natural resources of earth. On the other hand, there is an opinion that global annual GHG emissions will have to be cut by up to 80% (relative to 1990 levels) before 2050 to prevent the worst effects of climate change^2^. In order to arrive at the best package of practices for sustainable dairy farming, in recent years, there has been an increasing focus on evaluating the environmental effects of different milk production systems^3^. Though the environmental footprints of dairy production are calculated at national or provincial levels, there exist wide range of farming practices specific to farm at village level, which can potentially have varying ecological footprints when compared to national level. Hence, there is a need to evaluate environmental footprints of microcosmic and regional or farm specific production systems in order to arrive at best package of practices with greater promise of sustainability. An Irish estimate of pastoral based milk carbon footprints indicate that simple changes in management like quality of pasture, N application rates etc. can affect the total GHG emissions by 5–6% at both the farm and national levels^4^. In another Swedish study, Flysjö *et al*. (2011) proved that feed dry matter intake (DMI), N-fertiliser rate, N in excreta are among the ones that have largest impact on average milk carbon footprints with feed intake alone as critical factor since approximately 43% of the life-cycle GHG emissions of milk are due to feed cultivation including production and use of N fertilisers and diesel^5^.

Though most of the environmental footprint studies with respect to livestock production are related to cattle rearing, very few studies were conducted on environmental impact assessment of dairy buffalo production and it was estimated that about 5.07 kg of CO_2_ eq. are produced per kg of normalised buffalo milk, a value five times higher than that produced by dairy cow farms^6^. Another aspect of buffalo farming with a potentially high environmental impact is manure management^7^. Since emissions are often positively correlated with temperature, it is expected that tropical and subtropical ecosystems could produce greater N_2_O and CH_4_ emissions from animal excreta^8,9^. Further, the dietary protein content, feed digestibility, and sugar content are known to influence the amounts and types of N and C voided in cattle excreta^10^. Hence, considering the importance of feed related factors in ecological footprints of dairy bovine production, precise farm level quantification can enable farmers to pool resources and expertise, in taking necessary steps towards sustainable production. Even funding agencies, governments and NGOs increasingly recognize the benefits of taking a landscape scale approach to GHG quantification^11^. Since buffalo farming is one of the crucial activities in large parts of Asia, an action plan based on technical performance is needed to make it more profitable and sustainable. Besides, very meagre reports are available on quantification of GHG emissions and other environment attributes with respect to buffalo farming.

The main objective of this study is to assess the environmental sustainability of two different feeding regimens i.e., cottonseed meal and coated urea *vis a vis* production performance of dairy buffaloes. Feeding the milch buffaloes with cottonseed meal (CSM) alone or a mixture with CSM as a major constituent is customary in Indian subcontinent. However, CSM availability is seasonal coupled with unpredictability of cost apart from competition from other species farming like poultry and swine. In this regard, replacement of CSM by coated urea (CU), a non-protein-nitrogen (NPN) compound provides various advantages with respect to total cost for production, and CSM sparing effect for the meal usage in poultry, swine industry apart from being an indirect source of protein to animal. Since CSM replacement by CU is being viewed as one of the potential options due to above described reasons, we aim at comprehensive comparative analysis of the eco-sustainability trade-offs with respect to these two different feeding regimens.

## Results and Discussion

In a farming system, there is complex nature of the interactions between various components and it is essential to capture all relevant information for meaningful monitoring and evaluation of farming impacts on environment^11^. This experiment was intended to test the comparative effect of two different feeding regimens viz. CSM and SRU on the production parameters and environmental effects taking into account all the relevant information. The ingredient composition, nutrient composition and protein fractions of the diets fed are presented in Table 1.

**Table 1:** Ingredient and chemical composition of the rations fed to the experimental animals.

Item	Trial I		Trial II	
	CSM	SRU	CSM	SRU
**Ingredient composition of diet (g/Kg)**				
Hybrid Napier	*Ad libitum*	*Ad libitum*	—	—
Corn Stover	—	—	700	700
Maize grain	305.0	500.0	91.5	150.0
De-oiled rice bran	315.0	300.0	94.5	90.0
Cotton seed meal	300.0	100.0	90.0	30.0
Sesame meal	50.0	50.0	15.0	15.0
SRU (Optigen II)	0	20.0	0.0	6.0
Mineral mixture^1^	20.0	20.0	6.0	6.0
Sodium Chloride	10.0	10.0	3.0	3.0
**Nutrient composition of concentrate mixture/Complete feed (g/Kg)**				
DM	896.1	894.1	911.2	910.6
OM	894.2	904.7	898.2	903.3
CP	191.0	190.8	92.5	92.5
EE	31.0	28.0	25.2	21.9
TC	672.2	685.9	780.5	788.9
NFC	388.9	543.4	217.7	255.7
ADF	175.7	135.3	361.2	348.5
NDF_ap_	283.3	214.7	562.9	555.0
Hemicellulose	140.1	106.6	255.3	254.3
Ca^2^	15.6	15.1	4.9	4.8
P^2^	6.2	5.5	3.1	2.6
**Protein fragments of concentrate mixture/Complete feed (%)**				
Fraction PA^3^	12.35	19.63	10.06	12.04
Fraction PB1^3^	14.57	15.95	8.90	9.52
Fraction PB2^3^	51.63	44.55	36.00	33.88
Fraction PB3^3^	11.41	10.70	22.95	22.74
Fraction PC^3^	10.04	9.17	22.09	21.83
RDP (g/Kg CP)^4^	405	510	520	610
UDP (g/Kg CP)^4^	595	490	480	390

DM - Dry matter; OM - Organic matter; CP - Crude protein; EE - Ether extract; TC - Total carbohydrate; NFC - Non fiber carbohydrate; ADF - Acid detergent fiber; NDFap - Neutral detergent fiber corrected for ash and protein; Ca - Calcium; P - Phosphorus; PA - Protein A; PB1 - Protein B1; PB2 - Protein B2; PB3 - Protein B3; PC - Protein C; RDP - Rumen degradable protein; UDP - Undegradable protein.

^1^Mineral mixture contains 200 g of Ca, 60 g of P, 60 g of Na, 30 g of K, 20 g of Mg, 20 g of S, 3000 mg of Zn, 15000 mg of Mn, 650 mg of Cu, 650 mg of Fe, 40 mg of I, 20 mg of Se, 10 mg of Cr, 2,00,000 IU of Vitamin A, 50,000 IU of Vitamin D, and 1500 IU of Vitamin E.

^2^Sum of proportion obtained from individual feed ingredients and Mineral mixture.

^3^Sum of proportion of each ingredient’s protein fractions.

^4^Calculated as per the standard values of feed ingredients^50^.

### Damage to coated urea

**Figure 1:**
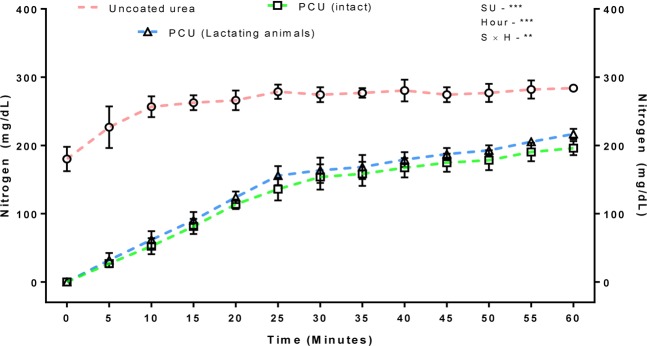
Nitrogen release with reference to the source of urea and hour of sampling (Shown as means and standard errors of triplicate incubation). PCU (intact) – Polymer coated urea from manufacturer; PCU (Lactating animals) – Polymer coated urea picked up from concentrate mixture); SU – Source of urea; S × H – Source × Hour interaction; ***P < 0.001; **P < 0.01

The N release rates were altered (P < 0.001) by both source of urea and hour of sampling (Fig. 1). Significant (P < 0.01) interactions were found between source of urea and hour of sampling. The urea readily dissolves in rumen liquor and the nitrogen release from polymer-coated urea (PCU) occurs steadily through the polymer coating. Accordingly, the PCU possesses advantage over uncoated urea by preventing the surge of N release into rumen environment. Nevertheless, damage to the coating may decrease the effectiveness of PCU due to the faster N release rates. At end of the *in vitro* experiment, the N release from the PCU granules picked up from the concentrate mixture fed to lactating buffaloes was 76.29% as much as uncoated urea (Supplementary Table 1). The intact PCU collected directly from manufacturer released 69.01% of N compared to that of uncoated urea in 1 hour of incubation. The altered N release rates of the PCU collected from concentrate mixture to those of intact urea revealed a significant damage occurred to polymer coating. Likewise, in our previous study, we have noticed a considerable damage to the outer coating of PCU isolated from total mixed ration^12^. The damage to the polymer coating may occur due to the blending or mixing of PCU with other feed ingredients while preparing either concentrate mixture or complete feed^12^.

### Milk yield and composition

Milk yield is one of the most influential parameters of environmental footprint estimates^13^ and increasing the dairy animals’ productivity by adopting various feeding strategies is one of the ancillary methane mitigation techniques^14^. In our study, although not significant, the milk yield in the SRU fed group was increased by 4.41% per day compared to CSM fed buffaloes. The milk components were not affected by either diet or pre-partum dry period, however, time had a significant influence on milk yield and all milk components, except for total protein yield (Table 2). These results are in accordance with the previous reports, which reported unaltered milk yield and components with varied prepartum dry period lengths^15^. Lactose is the most constant constituent of milk, which cannot be subjected large changes through nutritional manipulation^16^, and is neither altered in the present study. Further, it is noteworthy that the solids not fat content predominantly depends upon altering lactose and protein proportions of the milk^16^, which were not different between the two groups. By evaluating the milk record, it could be comprehended that altered RUP levels did not modify milk components. However, Nisa *et al*. (2008) reported a higher (P < 0.05) milk protein by decreasing the rumen degrading protein (RDP) content in diet of Nili Ravi buffaloes^17^. These inconsistencies could be due to the alterations in breed, feed ingredients, and level of RDP to RUP ratio. The slightly improved feed efficiency observed in lactating graded Murrah buffaloes fed rations supplemented with SRU might be attributed to the improved milk yield. Improved feed utilization efficiency decreases the total organic matter requirement to be fed thereby reducing carbon footprints of milk. With respect to income over feed cost per day (IOFC), the lactating buffaloes subjected to SRU diets showed better gain (0.38 $/d) compared to CSM fed group. Further, no interaction (P > 0.05) was found among diet, pre-partum dry period and time (week) on the milk yield and milk components.

**Table 2:** Milk yield and composition of lactating Murrah buffaloes fed diets containing cotton seed meal and slow release urea.

Item	Diets		DP Management		SEM	*P* Value		
	CSM	SRU	Short DP	Long DP		Diet	Time	DP^1^
Milk Yield	14.51	15.15	14.89	14.77	0.84	0.466	0.003	0.850
Fat yield	1.07	1.12	1.08	1.11	0.05	0.378	0.010	0.847
Lactose yield	0.74	0.79	0.77	0.76	0.04	0.386	0.001	0.821
Total Protein yield	0.55	0.59	0.57	0.57	0.03	0.380	0.093	0.653
SNF yield	1.43	1.52	1.49	1.47	0.08	0.385	0.001	0.772
Total Solids yield	2.50	2.64	2.57	2.58	0.13	0.376	0.002	0.914
6% FCMY^2^	16.81	17.61	17.06	17.36	0.82	0.397	0.006	0.935
ECMY^3^	21.33	22.49	21.79	22.04	1.03	0.363	0.006	0.990
FPCM^4^	21.07	22.13	21.46	21.75	1.01	0.382	0.009	0.994
Fat	7.45	7.50	7.34	7.60	0.18	0.921	0.028	0.343
Lactose	5.11	5.21	5.19	5.12	0.04	0.202	0.026	0.388
Total Protein	3.84	3.93	3.88	3.88	0.11	0.954	0.806	0.430
SNF	9.90	10.07	10.03	9.95	0.10	0.419	0.827	0.161
Total Solids	17.35	17.57	17.37	17.55	0.82	0.980	0.452	0.290
DMI (Kg/d)	21.57	21.90	21.73	21.74	0.47	0.681	0.001	0.836
FCE^5^	0.78	0.80	0.79	0.81	0.03	0.310	0.254	0.873
FE_L_ (MJ/d)^6^	3.07	3.20	3.16	3.14	0.11	0.262	0.204	0.978
IOFC ($/d)^7^	6.33	6.71	6.55	6.49	0.46	0.464	0.001	0.905
CF/Kg 6%FCMY ($/d)^8^	0.20	0.19	0.20	0.19	0.01	0.448	0.034	0.650

^1^DP - Prepartum dry period; ^2^6% FCMY − 6% Fat corrected milk yield; ^3^ECMY - Energy corrected milk yield; ^4^FPCM - Fat and protein corrected milk yield; ^5^FCE (Feed conversion efficiency) = 6%FCMY/TDMI; ^6^FE_L_ (Feed efficiency for lactation) (MJ/Kg) = Milk NEL(MJ/d)/DMI(Kg/d); ^7^IOFC ($) - Income over feed cost; ^8^CF/Kg 6%FCMY ($) - Cost of feed per Kg 6% FCMY.

### Nutrient intake and digestibility coefficients

The second important parameter after milk yield that is a key determinant of milk carbon footprints is feed intake^13^. The intakes of dry matter (DM), organic matter (OM), crude protein (CP), and neutral detergent fiber corrected for ash and protein (NDFap) did not differ between the treatments; however, the non fiber carbohydrate (NFC) consumption tended to be higher (P = 0.074) in SRU fed group (Table 3). The unaltered DMI indicates the fact that SRU incorporation did not affect the palatability of the diet. The trend (P = 0.074) of increased NFC intake in SRU group could be explained by the higher NFC content in the group compared to control, which was due to incorporation of SRU in the concentrate mixture duly reducing the level of CSM along with increased maize incorporation to formulate the diets iso-nitrogenous. Decreased nutrient digestibility apparently reduces the conversion efficiency and henceforth the production, thus compromising the milk yield per unit DMI leading to more carbon footprints. In this regard, nutrient digestibility coefficients could be considered as a vital animal based indicators to analyse the animal-based environmental pollutant emissions. However, the incorporation of SRU did not affect the digestibility coefficients of various gross nutrients and cell wall components, except for CP, which tended to be increased (P = 0.095) on SRU incorporation. The positively tended (P = 0.095) CP digestibility coefficients in SRU group could be attributed to lesser chemical complexity of SRU^18^. This finding is consistent with those reported elsewhere^19^ that replacement of traditional protein source with NPN compounds tends or significantly improves the protein digestibility. In ruminants, the CP digestibility is influenced by the hydrolysis rate of the protein molecule, whose structural complexity decides the rate of hydrolysis and therefore digestibility^20^. No influence of dry period (DP) allotment was noticed on the intakes and nutrient digestibility. Conversely, a research claimed greater DMI and nutrient digestibility coefficients for shortened dry period cows^21^; however, the nutrients were checked for intake and digestibility coefficients three weeks post-partum, unlike the present study. Further, the analogous nutritive value of rations in terms of digestible crude protein (DCP), total digestible nutrients (TDN), metabolizable energy (ME), and DCP/ME could be associated with similar nitrogen portion, DMI and nutrient digestibility coefficients of the diets.

**Table 3:** Nutrient intakes and digestibility coefficients of lactating Murrah buffaloes fed diets containing cottonseed meal and slow release urea.

Item	Diets		DP Management		SEM	*P* Value	
	CSM	SRU	Short DP	Long DP		Diet	DP
DM	22.33	23.14	23.38	22.09	2.99	0.649	0.474
OM	19.97	20.76	20.94	19.80	2.69	0.620	0.476
CP	2.41	2.48	2.51	2.37	0.36	0.745	0.506
NDF_ap_	11.52	11.55	11.88	11.19	1.38	0.968	0.433
NFC	5.56	6.63	6.27	5.92	1.07	0.074	0.514
RDP	1.47	1.80	1.63	1.64	0.12	0.048	0.914
DM	59.87	60.90	61.43	59.34	1.41	0.644	0.361
OM	63.07	64.27	64.64	62.70	1.36	0.563	0.360
CP	63.68	66.40	65.53	64.55	1.79	0.095	0.721
EE	67.01	67.90	67.20	67.71	1.45	0.703	0.826
TC	62.87	64.04	64.53	62.38	1.59	0.627	0.383
NFC	82.90	83.07	83.07	82.89	1.64	0.950	0.945
ADF	46.57	47.19	48.94	44.82	1.29	0.769	0.179
Hemicellulose	61.88	62.60	62.71	61.76	1.80	0.771	0.704
NDFap	53.23	54.24	55.38	52.09	1.57	0.671	0.192
Dig. CP (g/Kg)	68.60	70.90	70.31	69.25	2.03	0.177	0.742
Dig. Nutrients (g/Kg)	582.10	595.40	597.32	580.19	12.64	0.493	0.380
Dig. CP (g)/ME (MJ)	7.82	7.88	8.01	7.69	0.22	0.897	0.434

DM - Dry matter; OM - Organic matter; CP - Crude protein; EE - Ether extract; TC - Total carbohydrate; NFC - Non fiber carbohydrate; ADF - Acid detergent fiber; NDFap - Neutral detergent fiber corrected for ash and protein; Dig. CP - Digestible crude protein; DMI - Dry matter intake; ME - Metabolisable energy.

### Energy balance

**Figure 2:**
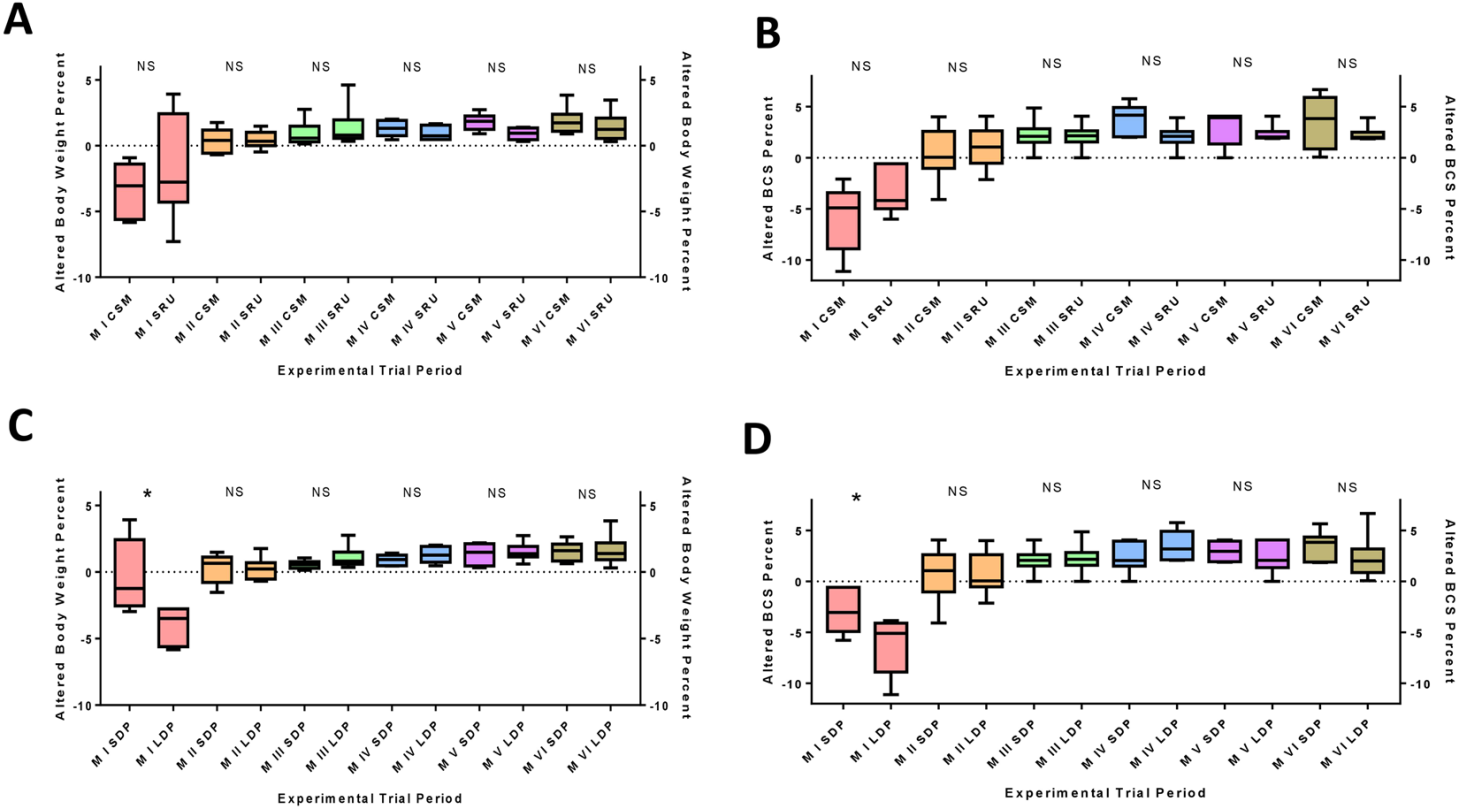
Altered BW and BCS with reference to diets fed and prepartum dry period lengths allotted. (**A**) ▲BW with reference to diet changes. (**B**) ▲BCS with reference to diet changes. (**C**) ▲BW with reference to Prepartum dry period changes. (**D**) ▲BCS with reference to Prepartum dry period changes. CSM - Cotton seed meal; SRU – Slow release urea; SDP – Short dry period; LDP – Long dry period; M – Month. NS – No Significance; *P ≤ 0.05.

The altered body weight (▲BW) and body condition score (▲BCS) percentages were not affected by SRU incorporation, however, the animals subjected to short dry period lengths showed a less (P < 0.05) BW loss compared to those of long dry period lengths (Table 4). The monthly changes revealed that both SRU incorporation and dry period allotment did not affect BW and BCS for entire experimental period, except for first month, which were positively altered by short pre-partum dry period allotment (Fig. 2A–D). The optimum BW and BCS are essential for maintaining optimum energy balance besides preventing various metabolic disorders. A tendency of interaction (P = 0.098) among diet, period, and prepartum dry period was observed for BCS. Although the NE balance was statistically similar between the two groups, it was higher by 81% in short DP group. The high NE balance reduces the risk of metabolic disorders such as ketosis, retention of placenta, and downer cow syndrome^22^. These results can be ascribed to the superior microbial function in short DP animals. In case of reduced dry period allotment, the animals are exposed to high energy concentrate diets, thus ensuring a continuous adaptability of rumen microbes to the concentrate feed, which may further decrease the nutritional stress of sudden ration changes^23^. The high NE in short DP group might be directly related to the high serum glucose levels and reduced lipolysis^24,25^. The similar results of shortened dry period on physical indicators of energy reserves mobilization (BW and BCS) were reported earlier^22^. In connection to the increased NE and decreased BW loss in short DP animals, our study projects the shortened pre-partum dry period as an eco-friendly dairy management technique as milk yield/unit DMI will be higher. The positive energy balance in all the experimental animals revealed a gain in body reserves for next lactation. Further, substantial interactions (P < 0.05) were also found among the diet, time, and prepartum dry period for NEm output.

**Table 4:** Body weight, Body Condition Score, and Energy balance of lactating Murrah buffaloes fed diets containing cottonseed meal and slow release urea.

Item	Diets		DP Management		SEM	*P* Value		
	CSM	SRU	Short DP	Long DP		Diet	Time	DP^1^
Body Weight	621.88	618.33	625.45	614.76	4.55	0.406	0.001	0.067
Body Condition Score	3.10	3.09	3.16	3.03	0.05	0.546	0.023	0.229
▲BW	0.50	0.52	0.72	0.30	0.58	0.915	0.001	0.046
▲BCS	1.18	1.08	1.44	0.81	1.21	0.825	0.001	0.173
NE_L_ Intake	123.89	128.28	127.88	125.52	4.90	0.501	0.001	0.580
NE_M_ Output	54.59	54.58	54.57	54.59	1.08	0.241	0.001	0.269
NE_L_ Output	66.45	70.10	67.90	68.85	3.24	0.360	0.008	0.989
NE Balance	2.85	3.61	5.41	1.04	1.49	0.766	0.303	0.119

^1^DP - Prepartum dry period; ▲BW - Altered Body Weight Percent; ▲BCS - Altered Body Condition Score Percent.

### Nitrogen dynamics

Nitrogen dynamics is a critical aspect in overall carbon footprint calculations since excess nitrogen is often associated to high GHG emission per functional unit (FU), indicating low efficiency of feeding and resource utilization^26^ apart from its potential to increase N_2_O and NH_3_ emissions^27^. Since, SRU is a NPN compound, any attempt to use it as a replacement for CSM is touted with the possibility of higher N excretion, an undesirable feature for sustainable dairying. However, in our study, we proved that the excretion rates of N through urine, faeces or milk were not affected by both the altered diet and dry period length (Table 5). By analysing the positively tended (P = 0.095) CP digestibility coefficient in parallel to the increased (P < 0.05) SUN concentration at 3 hr post-feeding, it is evident that the SUN was recycled through saliva across the ruminal wall, rather excretion through urine. This situation further reflects the fact that the SRU, although damaged to a little extent, was able to release the NH_3_-N at substantial levels without overloading the ability of liver to metabolise it, thus increasing the microbial protein yield and total N outflow to the intestine. As hypothesized, feeding SRU diet, though insignificant, decreased the faecal N loss without altering urinary N loss, consequently providing more N towards Milk and body weight regain besides abating the atmospheric N emission. The conversion efficiency of dietary N into milk N was 8.96% higher (P < 0.01) in SRU group compared to control. Although the SRU diets possess higher fraction of degradable N, no change in N excretion was witnessed revealing an unaltered N utilization efficiency on replacing CSM with SRU. This phenomenon further explains the ability of coated urea in releasing the nitrogen slowly within the threshold limit of bacterial N usage. Furthermore, the milk urea N, a proxy indicator of environmental performance and commonly used industry tool to fine-tune concentrations of dietary N and energy, did not affect with SRU incorporation revealing the optimum N and energy synchronization for both diets within the rumen^28^.

**Table 5:** Dietary Nitrogen partitioning among the experimental buffaloes with altered feed and pre-partum dry period.

Item	Diets		DP Management		SEM	*P* Value	
	CSM	SRU	Short DP	Long DP		Diet	DP
Total N Intake (g/d)	385.54	396.30	402.08	379.76	23.34	0.745	0.506
Degradable N Intake^1^	235	288.63	266.97	256.64	19.29	0.033	0.563
Faecal N (g/d)	140.83	132.29	138.91	134.20	10.97	0.590	0.765
Urinary N (g/d)^2^	136.27	137.43	137.65	136.05	7.51	0.921	0.842
Manure N (g/d)^3^	277.10	269.72	276.56	270.25	14.79	0.714	0.754
Milk N (g/d)^4^	78.51	88.30	84.83	81.98	5.68	0.216	0.705
CEDN^5^	20.33	22.33	21.17	21.49	0.62	0.026	0.674
Total N Outgo (g/d)	355.61	358.02	361.40	352.23	19.98	0.928	0.732
N balance (g/d)	29.93	38.29	40.68	27.53	7.50	0.533	0.335
apDN^6^	244.71	264.02	263.17	245.56	17.13	0.432	0.472
apMN^7^	108.44	126.58	125.52	109.51	11.14	0.336	0.393
Serum Urea N (Before feeding) (mmol/L)	5.98	6.01	6.05	5.94	0.19	0.940	0.715
Serum Urea N (3 hr post-feeding) (mmol/L)	6.37	7.07	6.87	6.57	0.26	0.045	0.347
Milk Urea N (mmol/L)	2.98	3.04	3.00	3.02	0.15	0.834	0.947

^1^Degradable N Intake = (RDP intake from H.Napier + Concentrate)/6.25.

^2^Urinary N (g/d) = 0.026 × BW × MUN (mg/dL)^59^.

^3^Manure N = Faecal N + Urinary N.

^4^Milk N = Milk Protein ÷ 6.38.

^5^CEDN; Conversion efficiency of dietary N = Milk N × 100 ÷ N Intake.

^6^Apparently digested N (apDN) = N intake – Faecal N.

^7^Apparently metabolized N (apMN) = apDN – Urinary N.

### Rumen fermentation parameters

**Figure 3:**
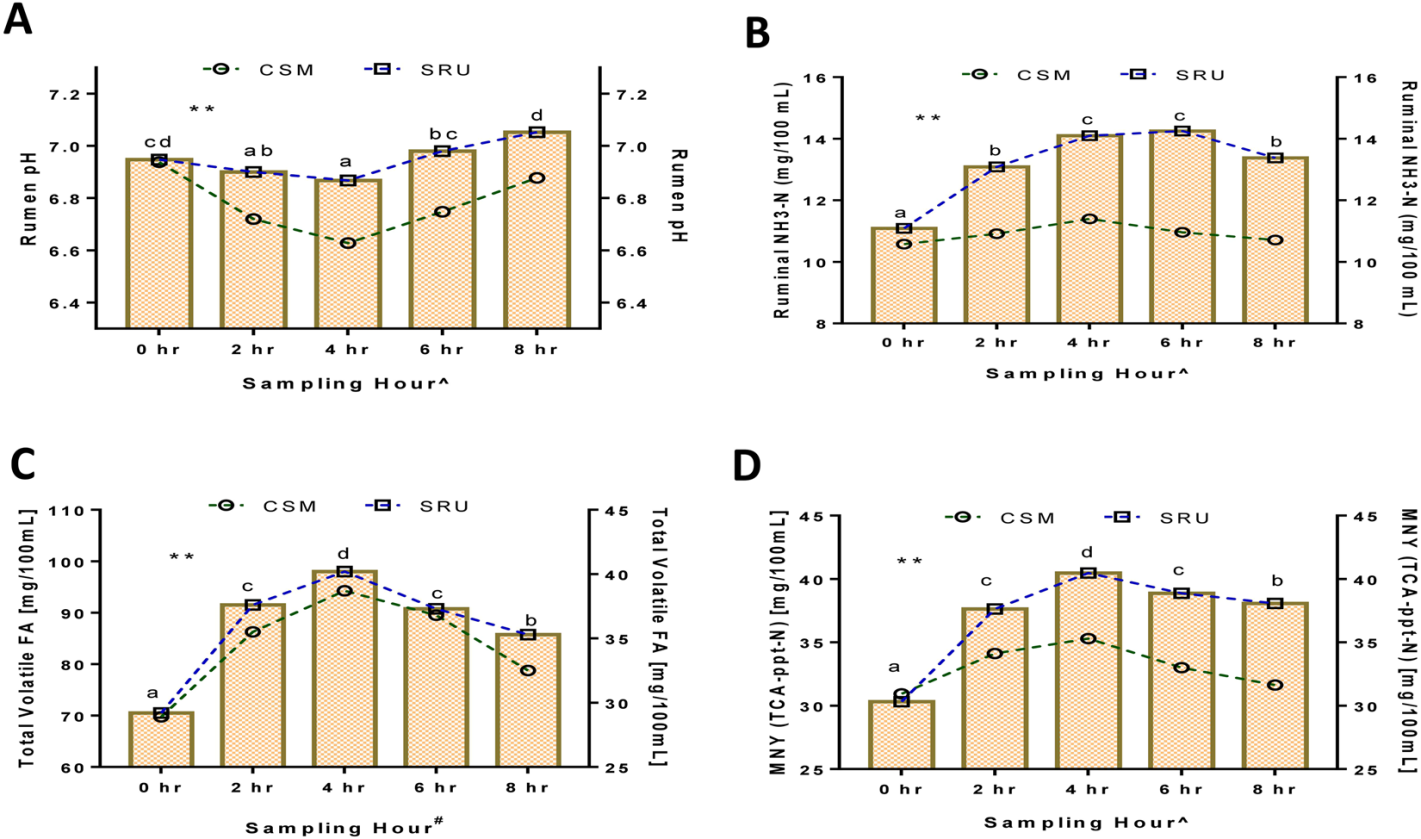
Dynamics of rumen pH, NH_3_-N, TVFA, and MNY content with reference to the diet change at various sampling periods. (**A**) Dynamics of post-prandial Rumen pH. (**B**) Dynamics of post-prandial Rumen NH_3_-N. (**C**) Dynamics of post-prandial Total Volatile Fatty acids. (**D**) Dynamics of post-prandial Microbial N yield. ^abcd^ Bars bearing different superscripts differ significantly; **P < 0.01; ^Diet × Hour interaction (P < 0.05); ^#^Tended to interact (P = 0.061).

**Figure 4:**
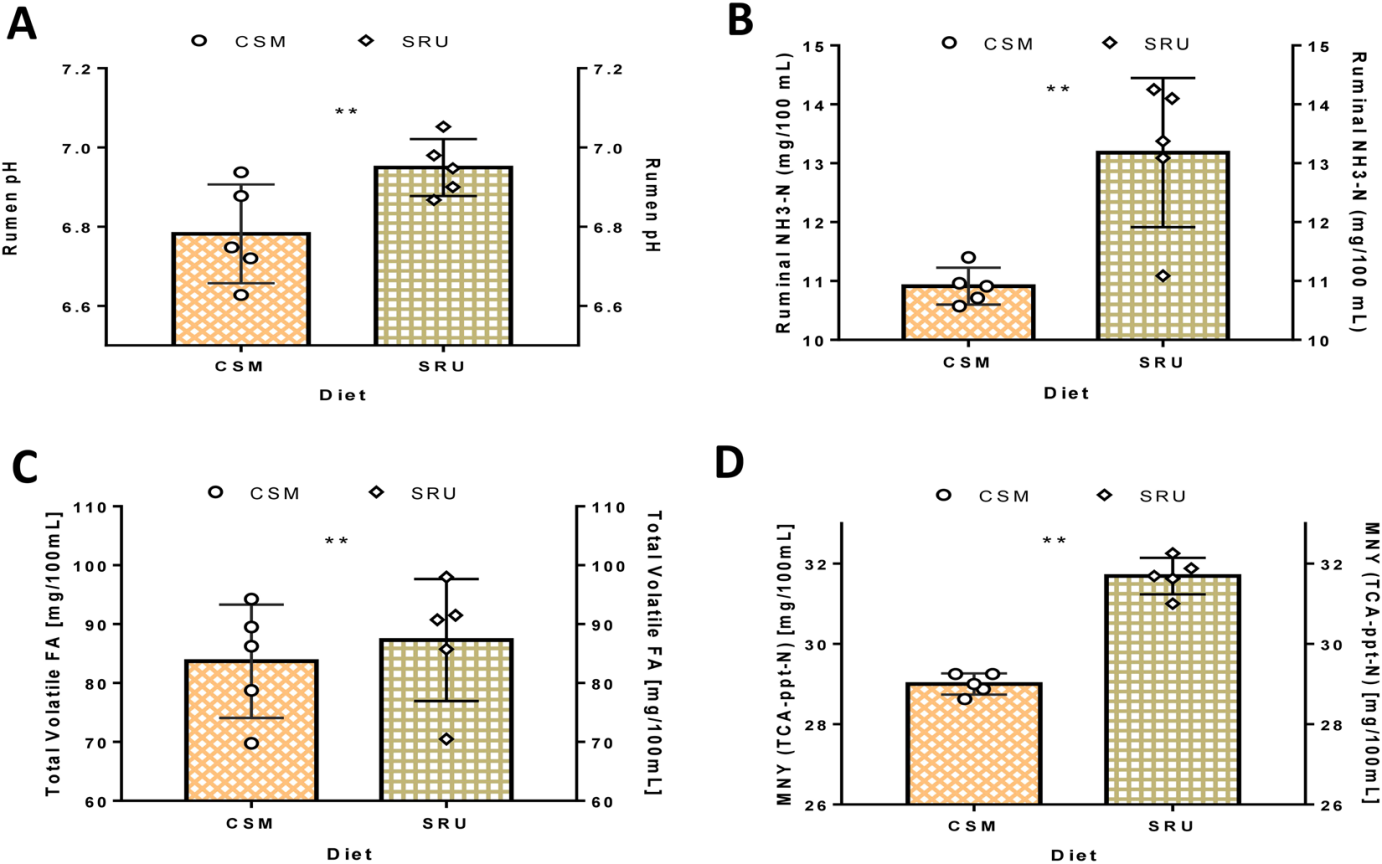
Mean rumen pH, NH_3_-N, TVFA, and MNY content with reference to the diet change. (**A**) Mean Rumen pH with reference to the diet change. (**B**) Mean Rumen NH_3_-N with reference to the diet change. (**C**) Mean Total Volatile FA with reference to diet change. (**D**) Mean Microbial N yield with reference to the diet change. **P < 0.01.

The SRU showed positive effect on rumen fermentation patterns including ruminal pH, total volatile fatty acids (TVFA), ammonia nitrogen (NH_3_-N), and Microbial N yield. Any adverse effects on these parameters could indirectly decrease the efficiency and production with a consecutively increased contribution to the menace of global environmental change. The dynamics of ruminal pH (Fig. 3A) is determined by the TVFA to NH_3_-N ratio rather NH_3_-N or TVFA alone, as proposed by Kim *et al*. (2014)^29^. The post-prandial increase (P < 0.01) in NH_3_-N, TVFA, and Microbial N concentrations (Fig. 3–D) might be due to sustained N release from coated urea, thereby facilitating its incorporation by rumen fauna for microbial protein synthesis and organic acids production. Significant Diet × Hour interactions were noticed among the pH, NH_3_-N, and Microbial N yield. The fiber degrading bacteria entirely depends upon the ammonia N for microbial protein synthesis, which increases the fibrolytic microbial load, thus surging cellulolytic and total bacterial counts in the groups fed with NPN compounds^30^. This notion explains that the improved Microbial N yield was due to a parallel increase in NH_3_-N content, which was persistent up to 8^th^ hour post-feeding. Besides, the increased proportion of fraction PA in treatment diet could have promoted more readily available N in the rumen. The beneficial effects of SRU on rumen fermentation profile is shown in Fig. 4.

### Livestock allied environmental attributes

Apart from the total and fixed solids, emissions of odorous volatile solids from livestock manure are of increasing environmental concern because of their ability to increase feedlot nuisance, if not managed properly^31^. In our study, replacing CSM with coated urea did not statistically affect the three faecal solid fractions (Table 6). The improved phosphorus utilization by rumen microbes might have caused a better phosphorus digestibility and its retention into milk, thus decreasing the faecal phosphorus (P) content^32^. The decreased faecal phosphorous excretion by buffalo bulls fed SRU diet could also be directly related to salivary phosphorous, which is supposed to diminish on decreasing dietary phosphorous intake^32^. The aforementioned statement reveals that high phosphorus content in CSM diet might be one of the reason for increased P excretion. Moreover, Alkaline diets cause an increased serum or urine pH that may combat the excretion of calcium (Ca), whose levels in excess causes acid precipitation further altering the chemistry of soil, therefore the plant growth and water quality in numerous ways^33^. Although little research on the influence of alkaline diets on mineral resorption is made in humans^33^, no data are available in describing the effects of NPN compounds incorporation on direct mineral reabsorption in ruminant species, which has to be explored. Altogether, these results propose that the herbivorous diets’ RDP percent and mineral content are the main factors that affect the excretion rate of certain pollutants like N, Ca, and P.

**Table 6:** Livestock allied environmental attributes of feeding and managemental regimen followed in the present study.

Item	Diets		DP Management		SEM	*P* Value		
	CSM	SRU	Short DP	Long DP		Diet	Time	DP
Residual Feed Intake^1^	0.17	−0.04	0.29	−0.17	0.29	0.491	0.001	0.643
Faecal Total solids (Kg/d)	8.62	8.55	8.36	8.82	0.23	0.860	NA	0.262
Faecal Volatile solids (Kg/d)	7.93	7.82	7.66	8.09	0.22	0.753	NA	0.263
Faecal Fixed solids (Kg/d)	0.69	0.74	0.70	0.73	0.05	0.520	NA	0.679
Faecal N (g/100 g faeces)	9.89	9.19	9.01	10.07	0.54	0.454	NA	0.751
Faecal Ca (g/100 g faeces)	63.67	64.14	61.13	66.68	4.34	0.934	NA	0.149
Faecal P (g/100 g faeces)	24.91	19.57	22.15	22.32	1.89	0.040	NA	0.875
Milk Ca (mg/dL)	180.94	184.75	183.72	181.97	6.30	0.679	NA	0.161
Milk P (mg/dL)	103.91	102.25	101.11	105.04	3.39	0.774	NA	0.603
MREDC^2^	21.50	24.58	23.33	22.76	0.90	0.003	NA	0.047
MREDP^3^	29.53	34.95	31.72	32.76	1.52	0.005	NA	0.491
Water Intake^4^	138.70	142.88	139.73	141.84	8.92	0.730	NA	0.861
Water/6% FCMY	6.21	6.18	5.97	6.41	0.17	0.921	NA	0.135
Faecal Lignin (%)	16.45	16.30	16.22	16.53	1.37	0.871	NA	0.735
Faecal Sand (%)	6.63	6.87	6.73	6.77	0.51	0.504	NA	0.921

NA – Not applicable.

^1^Calculated according to Cohen-Zinder *et al*. (2016)^60^.

^2^MREDC (Milk retention efficiency of dietary Calcium) = Milk Ca × 100 ÷ Ca Intake.

^3^MREDP (Milk retention efficiency of dietary Phosphorous) = Milk P × 100 ÷ P Intake.

^4^Water intake = Voluntary water intake + Moisture content of forage and concentrate mixture intake.

Further, the milk retention efficiency of dietary Ca and P contents was higher (P < 0.01) in SRU group, presumably due to the numerically improved milk yield in the SRU fed animals compared to those fed CSM feed. The improved Milk retention efficiency of dietary Ca (MREDC) and Milk retention efficiency of dietary P (MREDP) is a preferable outcome, because they may improve the efficiency of Ca and P utilization through increased Ca and P incorporation in milk, thereby reducing the environmental burden^34^. Hence, in this regard, feeding SRU could be considered as environmental friendly since nutritionists always advocate for practices that reduce P losses from farms without impairing profitability. Our study also reported a non-significant difference in Residual feed intake (RFI) between two groups. Residual feed intake (RFI), a new criterion for feed efficiency, could be defined as difference between the witnessed and expected intake for a given metabolic body weight and live weight gain. Consequently, low residual feed intake aids in efficient use of environmental resources, which may likely impose a restriction on livestock related environmental pollutants including greenhouse gases^35^. Higher quantities of complex substances, such as lignin or sand, do not decompose readily in soils and therefore contribute to poor quality manure. However, the lignin and sand contents of the buffaloes’ faeces were not altered by the diets fed.

### Environmental impact

Environmental impact of the entire trial is presented in Table 7. International experts have opined that non-CO_2_ emissions such as CH_4_ and N_2_O are less expensive to mitigate than CO_2_ emissions^36^. Hence, we wanted to evaluate the enteric methane emissions of our feeding trial in order to ascertain any upward and downward fluctuations. In our study, the enteric methane emission was not affected (P > 0.05) by both the diet fed and dry period allotted. The CH_4_ and N_2_O emission was lower in the manure excreted from SRU and Short DP allotted group of buffaloes. Further, the lifecycle assessment of various feed ingredients used in the trial revealed a lower global warming potential by 0.88 CO_2_ e per litre milk production. The preparation of feed ingredients of treatment ration needed 65 and 0.25 m^3^ less water to produce one tonne feed and 1000 litre 6% fat corrected milk yield (6% FCMY). Employing SRU as feed ingredient spares 1.02 hectares land per tonne feed production.

**Table 7:** Total Environmental impact of feeding and managemental regimen followed in the present study (Methodology I).

Item	Diets		DP Management		SEM	*P* Value		
	CSM	SRU	Short DP	Long DP		Diet	Time	DP^1^
CH_4_ (Kg/d)^1^	0.50	0.52	0.51	0.51	0.01			
CH_4_ (MJ/d)^2^	20.23	20.52	20.34	20.44	0.41	0.681	0.001	0.836
CH_4_ (MJ/d) / 6% FCMY (litre/d)	1.24	1.19	1.22	1.21	0.04	0.375	0.020	0.872
Methane and Nitrous oxide emissions from manure (Kg)								
CH_4_	10.16	10.38	9.86	10.67	NA	NA	NA	NA
CH_4_/6% FCMY (100 litres)	2.36	2.29	2.27	2.37	NA	NA	NA	NA
N_2_O	1.55	1.51	1.54	1.53	NA	NA	NA	NA
N_2_O/6% FCMY (100 litres)	0.36	0.33	0.35	0.34	NA	NA	NA	NA
Carbon foot print								
CFP_Feed_ (Kg CO_2_ e)^3^	20.74	17.29	19.38	18.57	NA	NA	NA	NA
GWP (Kg CO_2_ e)/6% FCMY	21.76	18.25	20.36	19.65	NA	NA	NA	NA
Land utilised^4^	4.72	3.70	NA	NA	NA	NA	NA	NA
Virtual water (m^3^)								
Virtual water/tonne feed	1062	997	NA	NA	NA	NA	NA	NA
Virtual water/ 1000 litres 6% FCMY	4.62	4.37	4.57	4.42	NA	NA	NA	NA

NA – Not Applicable.

^1^CH_4_ (Kg/d) = 18 + 22.5 × DMI (Kg/d)^61^.

^2^CH_4_ (MJ/d) = 1.29 + 0.878 × DMI (Kg/d)^62^.

^3^(Total DM consumed for 6 months × Fraction of GWP of individual feed ingredient)/Total 6% FCMY.

^4^Land required for the production of one tonne feed on dry matter basis (Hectares).

The feed consumed by lactating animals account for 86.35% of the total water footprint of milk^37^. Therefore, only feed accounts were employed for cradle to farm-gate lifecycle assessment in the present study. The SRU ration was dominated by the Maize fraction with a decreased percentage of CSM compared to control feed. Although the production of a tonne maize costs more water compared to cotton, usage of cotton industry byproduct (CSM) requires additional quantity of water because of the conversion factor and industry water requirement. For the same reason, the carbon footprint was higher for CSM compared to Maize dominated SRU diet (Table 7). The carbon footprint of feed ingredients’ production include the emission factors associated with preparation of fertilizers like urea apart from the direct emission from soil application. Therefore, adopting urea as a direct feed ingredient hold low carbon footprint compared to other conventional feed ingredients. Adopting coated urea as a replacer of CSM in buffaloes’ diets is environmental friendly, because of the lower GWP per litre milk production. Further, the questionnaire revealed higher weed removal along with herbicide and pesticide application rate for CSM compared to maize, which could exhibit more impact on environment in terms of climate change, ozone depletion potential, human health toxicity, aquatic ecotoxicity, terrestrial ecotoxicity, eutrophication potential and acidification potential.

**Figure 5:**
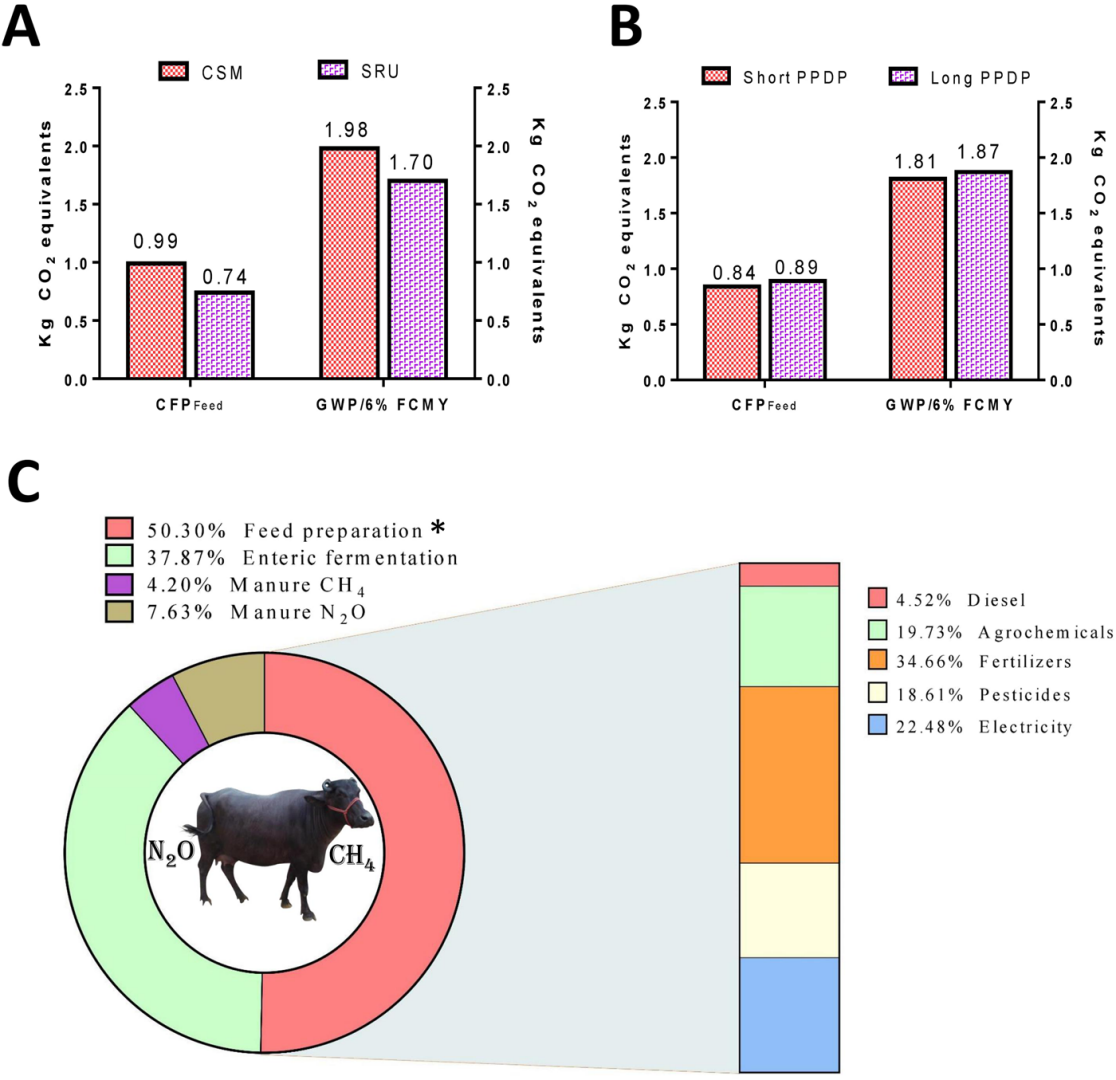
Share of individual global warming contributors, CFP_feed_, and GWP calculated according to methodology II. (**A**) CFP_feed_ and GWP/6%FCMY with reference to diet changes. (**B**) CFP_feed_ and GWP/6%FCMY with reference to prepartum dry period changes. (**C**) The share of individual global warming contributors. *Calculated through life cycle assessment (LCA) approach.

The life cycle assessment of the present trial also revealed that usage of agro-industrial byproducts causes more carbon footprint compared to those from direct agricultural output such as cereal or leguminous grains. Several inconstancies exist among the calculated carbon footprint and global warming potentialities in the cradle to farm gate LCA analysis. Such variations are principally attributed to the methodology used while estimating the GWP of feed, which confers a degree of uncertainty in the final GWP per liter 6% FCMY^12^. Therefore, we calculated the GWP by considering the emission intensities from byproducts as zero (methodology II). The CFP_feed_ and GWP per 6% FCMY with reference to the diet fed and prepartum dry period allotted are presented in Fig. 5A,B. The CFP_feed_ and GWP were higher in CSM diet- and long dry period- groups, irrespective of the methodology used. Between the two methodologies, including the feed byproducts instead of main feed ingredients tremendously increased the carbon footprint of feed production (avg. 19.02 vs 0.86 CO_2_ equivalents). The share of individual global warming contributors of the present trial is presented in Fig. 5C. Among different global warming contributors, feed preparation emitted highest share of CO_2_ equivalents (50.30%) followed by enteric fermentation (37.87%), manure CH_4_, and manure N_2_O (7.63%). In the group of GWP contributors during feed production, fertilizers imparted the highest proportion of CO_2_ equivalents (34.66%) followed by electricity (22.48%), agrochemicals (19.73%), pesticides (18.61%), and diesel (4.52%). These results are in complete agreement with the outcomes of previous research^12,38,39^.

**Figure 6:**
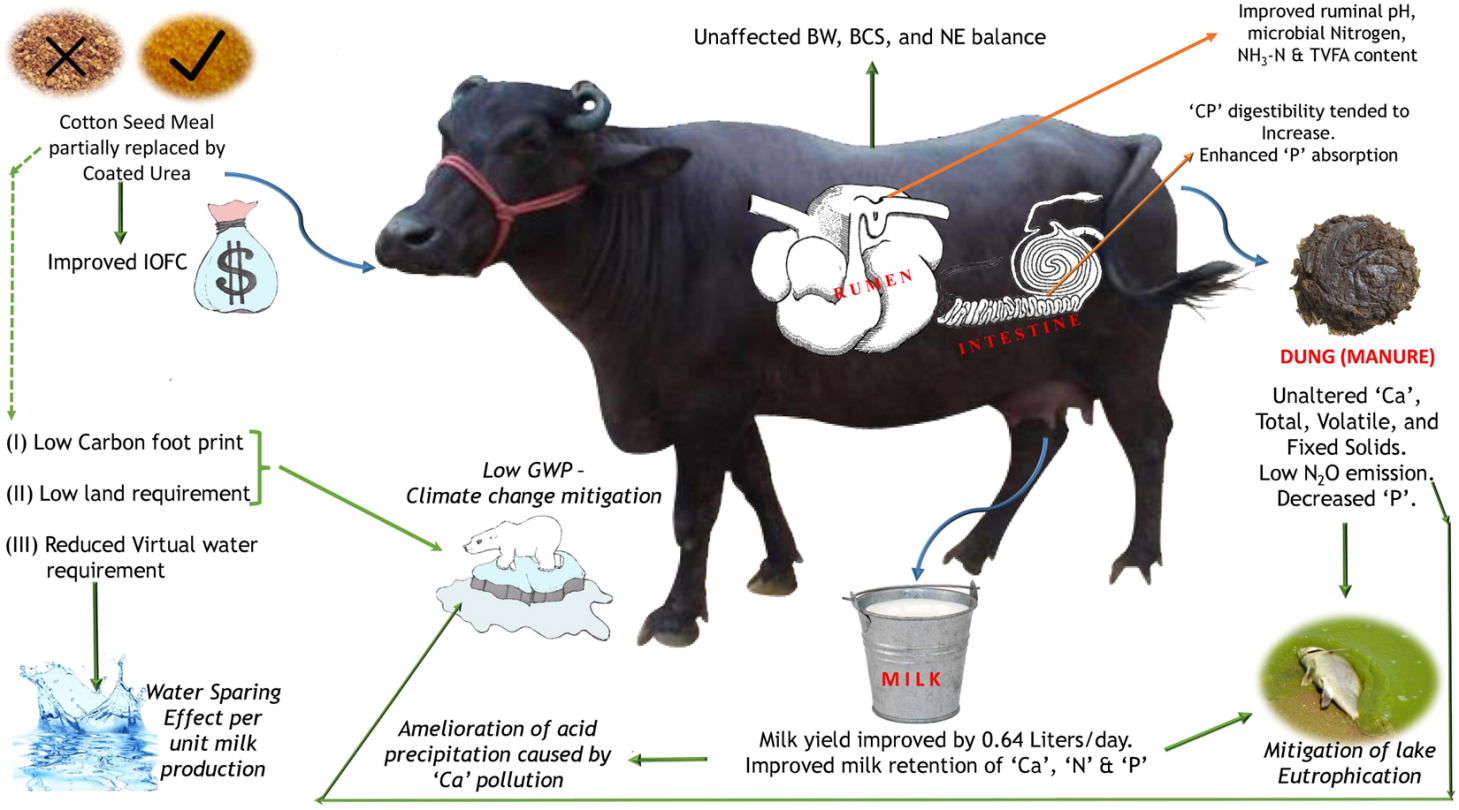
The potential benefits of cottonseed meal replacement with coated urea (Graphical Abstract).

In terms of virtual water, SRU production is linked with lower values of land requirement and virtual or embedded water when compared to CSM. If we consider at larger scales of production the lower values of land requirement and virtual water (VW) for SRU is highly encouraging to adopt it in the feeding management of livestock. Owing to rapid population growth coupled with climate change, there is going to be intense pressure on land and water resources of countries^40^. Hence adopting natural-resource efficient strategies will have conservational benefits in long run. The potential benefits of cottonseed meal replacement with coated urea are depicted in Fig. 6.

## Materials and Methods

All the experimental studies and corresponding protocols on the buffaloes were carried out in accordance with approvals of Institute’s animal ethics committee (IAEC), NTR College of Veterinary Science as per rules and guidelines framed and communicated by Committee for the Purpose of Control and Supervision of Experiments on Animals (CPCSEA), a statutory Committee, which is established under Chapter 4, Section 15(1) of the Prevention of Cruelty to Animals Act 1960, India.

### Slow release urea, animals, and experimental design

Among various sources of slow release urea (lignin- or CaCl_2_− or CaSO_4_− or Zinc- bound urea; and lipid- or polymer- coated urea), polymer coated urea (Optigen II; M/s Alltech Inc., Hyderabad, India) was selected for the present study (Supplementary Table 2). Coated urea is chosen among other slow release urea sources because of its ease of availability. For the trial I, twelve multiparous Murrah buffaloes were housed in well-ventilated and concrete floored shed, which were separated from one another by using partitions. Animals were divided into two groups, according to prepartum dry period lengths, days in milk, fat percent, body weights (BW), and body condition scoring (BCS) and stall fed throughout the experiment (Supplementary Table 3). For trial II, four graded Murrah buffalo bulls with average body weight 365.60 ± 14.92 kg were used in a replicated 2 × 2 Latin square design to reduce the influence of cofounding covariates. The four Murrah buffalo bulls were rumen cannulated a month before the trial as per the surgical procedure of laflin and gnad (2008)^41^.

### Damage to coated urea

The extent of damage to coated urea was measured by using the procedure given by Galo *et al*.^42^. The intact-coated urea granules before mixing into the concentrate mixture were used as negative control, whereas the normal (uncoated) urea granules were used as positive control. The granules of uncoated urea, intact polymer coated urea, and polymer coated urea in concentrated mixture were weighed (600 mg) and placed in beakers containing 100 ml of distilled water. The beakers were kept on automated mechanical shaker (RS-12R, Plate size: 7 × 11″). The liquid mixtures containing uniformly mixed N solute were sampled at 0 (immediately after incubation), 5, 10, 15, 20, 30, 40, 45, 50, 55, and 60 minutes. From each beaker, 5 ml was sampled and analysed for N concentration through enzymatic method-based diagnostic kits (M/s. ERBA Diagnostics Mannheim GmbH).

### Feeding management and collection of samples

Trial I - The lactating buffaloes were fed with *ad libitum* hybrid napier along with the concentrate mixture containing 0 (Control) and 20 (Treatment) g/Kg DM of slow release urea at 05:00 and 17:00 hours during the milking period. The trial was continued for a period of six months i.e., from first week of February to August. The experimental diets were isonitrogenous and formulated according to NRC (2001) recommendations as shown in Table 1. Milking was done twice a day (05:00 and 17:00 hours) and the milk samples were collected twice in a week for two consecutive days to estimate milk parameters. During the digestibility trial (at 19^th^ week), the feed was supplied and orts were weighed in the morning periods to calculate the total DM intake. The weekly milk samples throughout the experiment and the dung, feed, and orts collected during digestibility trial were frozen at −20 °C for later analysis. Further the animals were weighed monthly for body weights and the BCS was recorded by the same individual over six months trial period according to Alapati *et al*. (2010)^43^. The body condition scores were prioritized on a scale of 1 to 5 with 0.5 increment. These scores were established by characterizing the proportion of subcutaneous fat, which was felt by using 8 check points. The check points include; (i) Tail heat to pin bones, (ii) Spinous processes of the lumbar vertebrae (iii) Depression between the spinous and transverse processes (iv) Transverse processes of lumbar vertebrae (v) Point between 12^th^ and 13^th^ ribs (vi) Sacral crest (vii) Depression between sacral crest and hooks (viii) Depression between hooks and pins.

Trial II – The four buffalo bulls were allotted to one of two diets in a replicated 2 × 2 Latin square design, so that each animal is met with same feed twice during the trial. On day 21 of each rotation, rumen fluid was collected from cranial dorsal, cranial ventral, central rumen, caudal dorsal, and caudal ventral sites at 1 hour before feeding (0 h) and 2, 4, 6, and 8 h post-feeding. The rumen liquor were filtered by a four-folded muslin cloth with 250-µm pore size to avoid the undigested fiber material and debris. The rumen liquor is added with 20% trichloroacetic acid (1:5) to reduce ammonia volatilization and halt microbial activity. The samples were preserved at −20 °C for further analysis of rumen fermentation characteristics.

### Chemical analysis and calculations

Feed and faeces were subjected to proximate analysis (DM, CP, EE, and TA) as per the protocols prescribed by AOAC (2007)^44^. Nitrogen analysis was done using Turbotherm and Vapodest (Gerhardt, Germany) analyser. The total carbohydrates (TC) were calculated as per Sniffen *et al*. (1992)^45^:

1 $$TC=100-( \% CP+ \% EE+ \% TA)$$

Non fiber carbohydrates were estimated according to Hall *et al*. (1998)^46^:

2 $$NFC=100-[( \% CP- \% CPurea+ \% Urea)+ \% EE+ \% Ash+ \% NDFap]$$

wherein, CP = crude protein; CPurea = urea equivalent crude protein; EE = ether extract; and NDFap = neutral detergent fiber corrected for ash and protein. Cell-wall constituents were determined for feeds and faeces by using the methods described by Van Soest *et al*. (1991)^47^. Hemi-cellulose was calculated as NDF – ADF. The residual ash contents in NDF and ADF contents were estimated by ashing the samples in muffle furnace at 550 °C for 3 hours. Ash and protein corrected NDF in feed and faecal samples was estimated by using the equation:

3 $$NDFap=NDF-(NDIP+NDIA)$$

wherein, NDIP = Neutral detergent insoluble protein and NDIA = Neutral detergent insoluble ash. The residual N contents and protein fractions were estimated as per the procedures of Licitra *et al*. (1996)^48^ and Sniffen *et al*. (1992)^45^. Various solid fractions of the dung including total, volatile, and fixed solid portions were estimated as per the protocols of AOAC (2007)^44^. The average solid fractions of the entire digestibility trial was calculated as by employing the equation;

4 $$Total\,solids=\frac{(100-T{S}_{dig})\times Avg.DM{I}_{6m}}{100}$$

5 $$Volatile\,solids=\frac{(100-V{S}_{dig})\times Avg.DM{I}_{6m}}{100}$$

6 $$Fixed\,solids=\frac{(100-F{S}_{dig})\times Avg.DM{I}_{6m}}{100}$$

where, TS_dig_, VS_dig_, and FS_dig_ are the digestibility coefficients of total, volatile, and fixed solids, respectively.

A Lactoscan milk analyzer, (Model Lactoscan SL, Softrosys Technologies Pvt. Ltd, India) calibrated to the acceptable levels of Bureau of Indian standards (BIS) was used to analyse milk for composition including the fat, SNF, total protein and lactose. Milk urea nitrogen (MUN) and SUN values were estimated using diagnostic kit (M/s. ERBA Diagnostics Mannheim GmbH) following enzymatic method as mentioned by Talke and Schubert. The 6 percent fat corrected milk yield (FCM) was calculated as per Rice *et al*. (1970)^49^ as follows;

7 $$6 \% \,FCM=0.308\times Total\,Milk\,Yield\,(Kg)+11.54\times Total\,Fat\,Yield\,(Kg)$$

The total solids content of the milk was arrived by the addition of fat and SNF percentages. The Energy corrected milk (ECM) is calculated as per NRC (2001)^50^ by using the equation;

8 $$\begin{array}{rcl}ECM(Kg/d) & = & Milk\,yield(Kg/d)\times \{(0.0929\times  \% fat)\\  &  & +\,(0.0563\times  \% true\,protein)+(0.0395\times  \% lactose)\}/0.749\end{array}$$

The fat and protein corrected milk (FPCM) is calculated as per IDF (2015)^51^ as follows;

9 $$FPCM=Milk(Kg/d)\times [0.1226\times Fat \% +0.0776\times Protein \% +0.2534]$$

The milk samples of digestibility trial were subjected to Ca and P estimation using diagnostic kit (M/S. Autospan Diagnostics Ltd.) following OCPC method. Further, the Ca and P contents of feed and faeces were analysed by atomic absorption spectrophotometer. The pH of rumen liquor was measured immediately after collection using digital pH meter with a pH sensitivity of 0.01 units. The microbial N yield (MNY) was estimated by precipitating the trichloroacetic acid (TCA-ppt-N) as mentioned by Nandakumar *et al*. (2003)^52^. Ammonia-N was estimated by using Indophenol method^53^.

### Virtual water

The Virtual water requirement of the two rations was calculated by using the equation;

10 $$Virtual\,water\,(Lt)={\sum }_{(IFI)}\frac{W\times F{I}_{6m}}{CF\times 6 \% FCM{Y}_{6m}\times 1000}\,$$

where,

∑ _(IFI)_ – Sum of the fractions of individual feed ingredients.

W –Water requirement (m^3^/tonne output) [Calculated under Indian conditions as per Jayaram (2016)^54^].

FI_6m_ – Feed ingredient consumed for 6 months.

CF – Conversion factors to arrive the quantity of agricultural-byproducts used in the concentrate mixture fed (0.08 for Deoiled rice bran, 0.049 for Cottonseed meal, and 0.6 for Sesame meal).

6% FCMY_6m_ – 6% FCM yield for 6 months (Lt).

### Manure CH_4_ and N_2_O emission

The manure was stored in pit method and it was assumed that each day’s faeces was stored for a period of 180 days (trial period). The CH_4_ emission from Manure was calculated by using the following equation (Modified IPCC, 2006 Tier II Methodology^55^);

11 $$\frac{C{H}_{4}(Kg/animal)}{6 \% FCMY}=\frac{(100-V{S}_{dig})\times DM{I}_{6m}\times Boi\times 0.67\times (\frac{MCF}{100})}{6 \% FCM{Y}_{6m}\times 100}$$

where, VS_dig_ – Digestibility coefficient of Volatile solid;

12 $$V{S}_{dig}=\frac{V{S}_{intake}-V{S}_{outgo}}{V{S}_{intake}}\times 100$$

DMI_6m_ – Total dry matter intake for 6 months period

Boi – Maximum methane producing capacity (m^3^/Kg of VS) for buffaloes’ manure

0.67 – Conversion factor of m^3^ CH_4_ to Kg CH_4_

MCF – Methane conversion factor for pit method of storage at warm climate

6% FCMY_6m_ – 6% FCMY for 6 months.

The N_2_O emission from Manure was calculated by using the following equation (Modified IPCC, 2006 Tier III Methodology);

13 $$\frac{{N}_{2}O(Kg/animal)}{6 \% FCMY}=\frac{(100-percent\,{N}_{ret})\times N{I}_{tr}\times {N}_{2}O\,EF\times 44/28}{6 \% FCM{Y}_{6m}\times 100}$$

where,

$${N}_{retained}=(apMN\times 100)/{N}_{intake}$$

NI_tr_ – Total Nitrogen intake for the entire trial.

EF – Emission factor for solid storage.

44/28 – Conversion of N_2_O-N emissions to N_2_O emissions.

6% FCMY_6m_ – 6% FCMY for 6 months (Lt).

### Carbon footprint

The input data for carbon footprint calculation was composed according to the field interviews. The emission factors for farm inputs (fertilizers, on farm energy, and agrochemicals) were adapted from Adom *et al*. (2012)^56^, IPCC (2006)^55^, Audsley *et al*. (2009)^57^, and Deru and Torcellini (2007)^58^ (Supplementary Table 4).

Global warming potential (Modified IPCC, 2006 100a method^55^)

14 $$GWP=[(C{H}_{4m}+C{H}_{4En})\times 25]+({N}_{2}{O}_{m}\times 298)+{\sum }_{IFI}CFPFP$$

where,

CH_4m_ – Methane emission from manure (In Kg)

CH_4En_ – Enteric methane emission (In Kg)

N_2_O_m_ – Nitrous oxide emission from manure (In Kg)

IFI – Individual feed ingredient including roughage source

CFPFP – Carbon foot print for feed production (Kg CO_2_ equivalents)

The carbon footprint for feed production and total global warming potential were calculated according to two methods. In the first method, the emission intensity of feed was determined by calculating the emission from by-products, while the second method considered emission intensity of by-products as zero by assigning the whole emission to main products. The functional unit for virtual water usage, manure CH_4_ and N_2_O emission, and global warming potential was 1 litre 6% Fat corrected milk yield.

A flow chart of research work performed and definitions of various related technical terms used in the present study are described in Supplementary Fig. 1 and Supplementary Table 5, respectively.

### Statistical analysis

Trial I - Prior to the analysis, the daily recordings were averaged to weekly means. The Kolmogorov-Smirnov test performed to observe the distribution among various parametric variables revealed that all the parameters under study (milk yield, milk composition, and Body weight) followed a normal distribution. General Linear Model repeated measures (GLM-RM) analysis was applied to the data of milk yield, milk components, residual feed intake, and enteric CH_4_ emission considering the sampling day as repeated measure, with fixed effects of dietary treatments (D), sampling day as Week period (W), prepartum dry period (P) and the interactions among them (D×W×P) according to the model;

$${{\rm{Y}}}_{{\rm{ijkl}}}=\mu +{{\rm{D}}}_{{\rm{i}}}+{{\rm{W}}}_{{\rm{j}}}+{{\rm{P}}}_{{\rm{k}}}+{({\rm{D}}\times {\rm{W}}\times {\rm{P}})}_{{i}{\rm{jk}}}+{{\rm{A}}}_{{\rm{l}}}+{{\rm{e}}}_{{\rm{ijkl}}}$$

where Y_ijkl_ is the dependent variable, µ is the overall mean, D_i_ the effect of dietary treatment (i = 2), Wj the effect of sampling week (j = 26), Pk the effect of prepartum dry period (k = 2), (D × W × P)_*i*jk_ the interaction between dietary treatment, sampling week and prepartum dry period, A_l_ the animal’s random effect, and e_ijkl_ the residual error. The similar statistical procedure (GLM-RM) was also applied to the data of BW, ▲BW, ▲BCS, and net energy divisions with buffalo as a random effect and dietary treatment, sampling time, prepartum dry period and the interactions among them as fixed effects; but, the sampling day used as repeated measure in this context was month period (M). The model developed was;

$${{\rm{Y}}}_{{\rm{ijkl}}}=\mu +{{\rm{D}}}_{{\rm{i}}}+{{\rm{M}}}_{{\rm{j}}}+{{\rm{P}}}_{{\rm{k}}}+{({\rm{D}}\times {\rm{M}}\times {\rm{P}})}_{{i}{\rm{jk}}}+{{\rm{A}}}_{{\rm{l}}}+{{\rm{e}}}_{{\rm{ijkl}}}$$

where Mj the effect of recording month (j = 6), (D × M × P)_*i*jk_ the interaction between dietary treatment, recording month and prepartum dry period. All the data (milk yield, milk components, and body weight) estimated at the beginning of the experiment were used as covariates. The BCS between the subjects was confirmed for significance by means of Mann-Whitney U-test, whereas the statistical significance for BCS within the subjects (monthly changes) was calculated by Kruskal-Wallis H test. The nutrient intakes, digestibility coefficients of various gross nutrients and fiber fractions, cost economic parameters, milk retention efficiencies, solid fractions, manure emissions between the groups were tested for significance by using student’s t-test. Results are presented as mean values with the standard error of the means. Probability values with P ≤ 0.05 were considered significant and 0.10 ≥ P > 0.05 were considered as trend. Statistical analysis was performed by using SPSS (Version 23.0). No interactions were found among diet, time, and dry period for all the parameters, except for DMI (P < 0.01), BCS (tended to be significant), ▲BW (tended to be significant), and CH_4_ emitted (P < 0.05). Therefore, the interactions were presented in supplementary Table 6 (6a, 6b, 6c, 6d, 6e, and 6f) instead of the main tables.

Trial II - The hourly pH, NH_3_-N, TVFA, and MNY content was evaluated for statistical difference by adopting a GLM repeated measures analysis with hour as repeated measure and 0^th^ hour values as covariates. Post hoc analysis, wherever necessary is performed by marking least significant difference (LSD). All the graphs were generated by using GraphPad Prism 7.0 (GraphPad Software, Inc., San Diego, CA).

### Conclusion and recommendations

The study found that SRU as a replacement for CSM is advantageous both in terms of animal production and environmental attributes. In developing countries, any attempts to inspire farmers for environmental friendly practices require them to be easily adoptable and economical. The slow release urea as a replacement for cottonseed meal seems not only economical but also eco-friendly. Though the basic intention of farming is to maximize the yield from animals, it is also necessary to consider the environmental costs associated with it. Hence, feeding SRU as a source of protein will be cheaper alternative for optimal production within the genetic potential of animal. Replacing the conventional feedstuff with potential alternatives that can meet the nutritional demands of animal along with being sustainable in their utility is need of the hour. Hence, future studies should concentrate upon this aspect of animal farming especially in tropical production systems where future climate change is going to have considerable impact on livestock production.

## Supplementary information

Supplementary information

## Data Availability

The data related to the current study are available from the corresponding author on reasonable request.
