# Supplementary material for: Environmental sustainability assessment of tropical dairy buffalo farming vis-a-vis sustainable feed replacement strategy
Source: Sci Rep. 2019 Nov 14;9:16745. doi: 10.1038/s41598-019-53378-w (PMC6856187; doi:10.1038/s41598-019-53378-w)
Supplement: Supplementary file 1 — Supplementary information [file 41598_2019_53378_MOESM1_ESM.pdf]

Environmental sustainability assessment of tropical dairy buffalo farming vis-a-vis sustainable feed replacement strategy

P. Ravi Kanth Reddy[1], D. Srinivasa Kumar[2], E. Raghava Rao[3], Ch. Venkata Seshiah[4], K. Sateesh[5], K. Ananda Rao[6], Y. Pradeep Kumar Reddy[7], Iqbal Hyder*[8]

[1]Livestock Farm Complex, CVSc, SVVU, Proddutur – 516 360
[2]Dept. of Animal Nutrition, NTRCVSc, SVVU, Gannavaram – 521 102
[3]Administrative building, Sri Venkateswara Veterinary University, Tirupati – 517 502
[4]Livestock Farm Complex, NTRCVSc, SVVU, Gannavaram – 521 102
[5]AH Polytechnic college, SVVU, Banavasi – 518 323
[6]Buffalo Research Station, Venkataramannagudem – 534 101
[7]Centre for Continuing Veterinary Education and Communication, CVSc, Sri Venkateswara Veterinary University, Tirupati – 517 502
[8]Dept. of Veterinary Physiology, NTRCVSc, SVVU, Gannavaram – 521 102

Correspondence;
Dr. Iqbal Hyder
Iqbvet@gmail.com
Dept. of Veterinary Physiology, NTRCVSc, SVVU, Gannavaram – 521 102

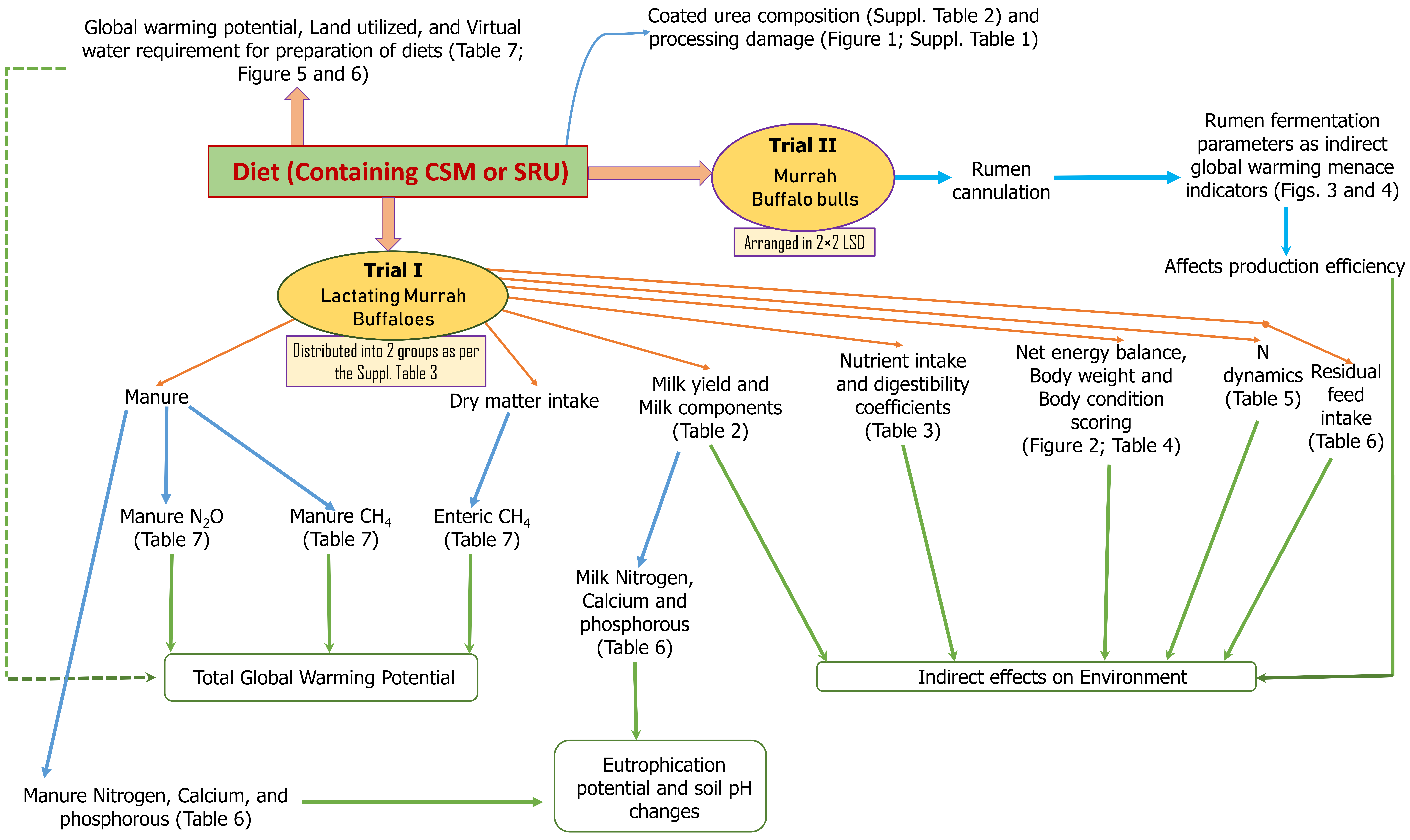

Supplementary Figure 1. Flow chart of the research work carried out in the present study

Supplementary Table 1. Mean values of the nitrogen released from different urea sources (n=3)

Hour	Uncoated urea	Intact PCU	PCU from lactating animals
0	180	0	0
5	226.67	27	32
10	256.67	52.33	61.67
15	262.67	81.33	90.33
20	266	113.67	124
25	278.67	136	155.67
30	274.33	153.67	163.67
35	277	158.33	168.33
40	280.33	167.33	179.33
45	274.33	174.67	187.33
50	277	178.33	192.67
55	282	190.33	205.33
60	284	196	216.67
N release (% of uncoated urea)	100	69.01	76.29

Supplementary Table 2. The composition of Coated Urea (g/100 g)

COMPONENT		DM	CPE	N	EE
Coated Urea	On DMB[1]	100	263.63	42.18	12.29
	"As is"[2]	99	261.00	41.76	-

[1]DMB – Dry matter basis
[2]As is – Fresh basis

DM – Dry matter, CPE – Crude protein equivalents, N – Nitrogen, EE – Ether extract

Supplementary Table 3. Distribution of graded Murrah buffaloes during lactation trial

	Animal No.	Body wt. (Kgs)	Prepartum Dry period	Lactation No.	Stage of Lactation (Days)	Milk Yield (Kg)	Fat (%)	6% FCM
**CSM**	230.00	600.00	75	3.00	48.00	18.80	7.40	21.84
	2652.00	597.00	82	3.00	32.00	13.59	7.50	15.95
	B – 3	709.00	45	3.00	26.00	19.67	6.80	21.49
	B – 9	614.00	64	3.00	21.00	17.87	7.10	20.14
	B – 21	583.00	42	3.00	24.00	12.81	6.80	13.99
	B – 20	654.00	43	3.00	20.00	19.42	6.90	21.45
	**Mean ± SE**	**626.17 ± 19.31**	**58.50 ± 7.18**	**3.00 ± 0.00**	**28.50 ± 4.27**	**17.03 ± 1.24**	**7.08 ± 0.12**	**19.15 ± 1.36**
**SRU**	761.00	585.00	45	3.00	46.00	15.85	7.60	18.78
	2662.00	570.00	40	3.00	30.00	16.12	7.10	18.17
	836.00	607.00	43	3.00	32.00	17.90	6.70	19.35
	B – 6	668.00	62	3.00	33.00	18.81	7.10	21.20
	B – 26	614.00	80	3.00	25.00	17.27	7.40	20.06
	B – 4	650.00	68	3.00	10.00	16.06	6.80	17.55
	**Mean ± SE**	**615.67 ± 15.30**	**56.33 ± 6.58**	**3.00 ± 0.00**	**30.17 ± 4.80**	**17.00 ± 0.49**	**7.12 ± 0.14**	**19.19 ± 0.54**

CSM – Cotton seed meal; SRU – Slow release urea (Coated Urea); 6% FCM – 6% Fat corrected milk yield.

Supplementary Table 4. Farm input (Fertilizers, Agrochemicals, and Energy) requirements and their emission factors

Farm inputs		Emission Factors
Fertilizer (Fe)	N	3.871 Kg CO_2 e/Kg N due to manufacturing of N fertilizer.
		0.633 Kg CO_2 e/Kg N due to field emissions CO_2.
		6.205 Kg CO_2 e/Kg N due to direct and indirect N_2O field emissions.
	P	3.028 Kg CO_2 e/Kg P due to manufacturing of P fertilizer.
	K	0.573 Kg Kg CO_2 e/Kg due to manufacturing of K fertilizer.
	S	3.855 Kg CO_2 e/Kg S in fertilizer.
Agrochemicals (A)	Lime	0.0158 Kg CO_2 e/Kg Lime due to manufacturing.
		0.4400 Kg CO_2 e/Kg $CaCO_3$ due to application on farm.
Pesticides (P)/Herbicides(H)/ Weedicides(W)*		Atrazine (188.3 MJ/Kg a.i), Trifluralin (150.9 MJ/Kg a.i), Pendimethaline (450 MJ/Kg a.i), Glyphosate (474 MJ/Kg a.i), Diuron (274.5 MJ/Kg a.i), Alachlor (277.5 MJ/Kg a.i)
Fuel (Fu)	Diesel	11.89 Kg CO_2 e/gallon.
Electricity (E)		0.653 Kg CO_2 e/KWh.
Requirements of Fe, A, P, H, W, Fu, and E for Hybrid Napier, Maize, cotton, rice, and Sesame		Questionnaire method (n = 30)

Synthesized from Adom et al. (2012), IPCC (2006), Audsley et al. (2009), and Deru and Torcellini (2007).
*Multiplied by conversion factor (0.069) to obtain Kg CO_2 equivalents.

Supplementary Table 5. Definitions of the various technical terms used in the manuscript

Technical Word	***Definition***
Slow release urea	Slow-release urea compounds are meant to release urea in a constant rate rather rapid.
Total carbohydrates	Total carbohydrates consist of multiple nutrients, including dietary fiber, sugars and starches.
Neutral detergent fiber (NDF)	NDF measures most of the structural components in plant cells (i.e. lignin, hemicellulose and cellulose), but not pectin.
Acid detergent fiber (ADF)	ADF measures the structural components lignin and cellulose in plant cells but not pectin.
Non fiber carbohydrates (NFC)	Include carbohydrates other than fiber
Total solids	Total solids is a measure of the suspended and dissolved solids in manure or any liquid.
Volatile solids	Volatile solids are those solids in water or other liquids that are lost on ignition of dry solids at 1,020°F.
Rumen liquor	The liquid obtained by squeezing and filtering the rumen contents.
TCA-ppt-N	Nitrogen, which gets precipitated in Trichloro-acetic acid (Microbial Nitrogen).
Volatile fatty acids	Microbes in the rumen ferment carbohydrates into volatile fatty acids (ATP contributors) which are absorbed through the rumen wall into the blood stream.
Fat corrected milk (FCM)	The milk standardized as per the fat percent.
Energy corrected milk (ECM)	The milk standardized as per the energy content.
Fat and protein corrected milk (FPCM)	The milk standardized as per the fat and protein percent.

Solids not fat	Milk solids-not-fat (MSNF) contain the lactose, caseins, whey proteins, and minerals (ash content) of the product from which they were derived. They are an important ingredient for the following beneficial reasons: improve the texture of ice cream, due to the protein functionality.
Virtual water	Virtual water trade (also known as trade in embedded or embodiedwater) refers to the hidden flow of water if food or other commodities are traded from one place to another.
Carbon foot print	The total amount of greenhouse gases produced to directly and indirectly support human activities, usually expressed in equivalent tons of carbon dioxide (CO_2).
Global warming potential	Global warming potential (GWP) is a measure of how much heat a greenhouse gas traps in the atmosphere up to a specific time horizon, relative to carbon dioxide.
Kolmogorov-Smirnov test	A nonparametric *test* of the equality of continuous, one-dimensional probability distributions that can be used to compare a sample with a reference probability distribution.
Body condition score	Is a visual assessment of the amount of fat and muscle covering the bones of a cow, regardless of body size
Manure CH_4	The methane produced from the decomposition of livestock *manure*
Manure N_2O	Nitrous oxide produced during the nitrification-denitrification of nitrogen contained in manure or animal faeces.
Digestion coefficient	The proportion of a nutrient taken into the digestive tract that is actually digested.
Rumen degradable protein (RDP)	The portion of dietary protein that can be degraded in the rumen, the largest of the multi-

	compartmental stomach, by microorganisms (both bacteria and protozoa) that use the protein to manufacture high quality microbial cell proteins, also known as microbial crude protein.
Rumen undegradable protein (UDP)	The protein, which is not degraded with in the rumen and gets directly absorbed at intestine without microbial manipulation.
Income over feed cost	Income over feed costs is defined as the portion of income from milk sold that remains after paying for purchased and farm-raised feed used to produce milk.
Net energy	Net Energy is a concept used in energy economics that refers to the difference between the total energy expended and the amount of energy gained.
Dry period	Dry period includes the time between halting of milk removal (milk stasis) and the subsequent calving.

Supplementary Table 6a. Milk yield and composition of lactating Murrah buffaloes fed diets containing cottonseed meal and slow release urea

Item	D×T×DP	D×T
Milk Yield	0.450	0.974
Fat yield	0.906	0.912
Lactose yield	0.403	0.974
Total Protein yield	0.862	0.967
SNF yield	0.698	0.970
Total Solids yield	0.797	0.949
6% FCMY	0.817	0.933
ECMY	0.860	0.937
FPCM	0.861	0.946
Fat	0.878	0.955
Lactose	0.992	0.841
Total Protein	0.069	0.890
SNF	0.903	0.661
Total Solids	0.415	0.937
DMI (Kg/d)	0.008	0.724
FCE	0.757	0.946
FE_L (MJ/d)	0.789	0.104
IOFC ($/d)	0.463	0.940
CF/Kg 6%FCMY ($/d)	0.947	0.867

D×T×DP – Diet×Time×Prepartum dry period interactions; D×T - Diet×Time Interactions; 6% FCMY – 6% Fat corrected milk yield; ECMY - Energy corrected milk yield; FPCM - Fat and protein corrected milk yield; FCM - Feed conversion efficiency; FE_L - Feed efficiency for lactation; IOFC – Income over feed cost; CF/Kg 6%FCMY – Cost of feed per Kg 6% FCMY.

Supplementary Table 6b. Nutrient intakes and digestibility coefficients of lactating Murrah buffaloes fed diets containing Cottonseed meal and slow release urea

Item	D×DP
DM	0.142
OM	0.143
CP	0.114
NDF_{ap}	0.174
NFC	0.184
RDP	0.400
DM	0.308
OM	0.255
CP	0.767
EE	0.984
TC	0.234
NFC	0.430
ADF	0.580
Hemicellulose	0.114
NDFap	0.248
Dig. CP (g/Kg)	0.791

Dig. Nutrients (g/Kg)	0.261
Dig. CP (g)/ME (MJ)	0.568

DM – Dry matter; OM – Organic matter; CP – Crude protein; EE – Ether extract; TC – Total carbohydrate; NFC – Non fiber carbohydrate; ADF – Acid detergent fiber; NDFap – Neeutral detergent fiber corrected for ash and protein; Dig. CP – Digestible crude protein; DMI – Dry matter intake; ME – Metabolisable energy.

Supplementary Table 6c. Body weight, Body Condition Score, and Energy balance of lactating Murrah buffaloes fed diets containing Cottonseed meal and slow release urea

Item	D×T×DP	D×T
Body Weight	0.584	0.114
Body Condition Score	0.098	0.122
▲BW	0.996	0.084
▲BCS	0.283	0.106
NE_L Intake	0.265	0.801
NE_M Output	0.040	0.148
NE_L Output	0.654	0.937
NE Balance	0.083	0.837

D×T×DP – Diet×Time×Prepartum dry period interactions; Diet×Time interactions; ▲BW – Altered Body Weight Percent; ▲BCS – Altered Body Condition Score Percent; NE_L – Net energy for lactation

Supplementary Table 6d. Dietary Nitrogen partitioning among the experimental buffaloes with altered feed and pre-partum dry period

Item	D×DP
Total N Intake (g/d)	0.925
Degradable N Intake	0.135
Faecal N (g/d)	0.114
Urinary N (g/d)[3]	0.129
Manure N (g/d)[4]	0.351
Milk N (g/d)[5]	0.093
CEDN[1]	0.873
Total N Outgo (g/d)	0.117
N balance (g/d)	0.089
apDN[6]	0.607
apMN[7]	0.221
Serum Urea N (Before feeding) (mmol/L)	0.300
Serum Urea N (3 hr post-feeding) (mmol/L)	0.758
Milk Urea N (mmol/L)	0.925

CEDN - Conversion efficiency of dietary N
apDN - Apparently digested N
apMN - Apparently metabolized N

Supplementary Table 6e. Livestock allied environmental attributes of feeding and managemental regimen followed in the present study

Item	D×DP
Residual Feed Intake[1]	0.290
Faecal Total solids (Kg/d)	0.951
Faecal Volatile solids (Kg/d)	0.901
Faecal Fixed solids (Kg/d)	0.340
Faecal N (g/100 g faeces)	0.258
Faecal Ca (g/100 g faeces)	0.121
Faecal P (g/100 g faeces)	0.268
Milk Ca (mg/dL)	0.593
Milk P (mg/dL)	0.827
MREDC[2]	0.382
MREDP[3]	0.537
Water Intake[4]	1.210
Water:6% FCMY	0.449
Faecal Lignin (%)	0.574
Faecal Sand (%)	0.634

MREDC - Milk retention efficiency of dietary Calcium
MREDP - Milk retention efficiency of dietary Phosphorous

Supplementary Table 6f. Total Environmental impact of feeding and managemental regimen followed in the present study

Item	D×DP[2]
CH_4 (MJ/d)	0.047
CH_4 (MJ/d) / 6% FCMY (litre/d)	0.098
CH_4	NA
CH_4/6% FCMY (100 litres)	NA
N_2O	NA
N_2O/6% FCMY (100 litres)	NA
CFP_{Feed} (Kg CO_2 e)[3]	NA
GWP (Kg CO_2 e)/6% FCMY	NA
Land utilised[4]	NA
Virtual water/tonne feed	NA
Virtual water/ 1000 litres 6% FCMY	NA

NA – Not Applicable
GWP – Global warming potential
CFP_{Feed} – Carbon footprint
